# A New Paradigm in Parkinson's Disease Evaluation With Wearable Medical Devices: A Review of STAT-ON^TM^

**DOI:** 10.3389/fneur.2022.912343

**Published:** 2022-06-02

**Authors:** Daniel Rodríguez-Martín, Joan Cabestany, Carlos Pérez-López, Marti Pie, Joan Calvet, Albert Samà, Chiara Capra, Andreu Català, Alejandro Rodríguez-Molinero

**Affiliations:** ^1^Sense4Care S.L., Cornellà de Llobregat, Spain; ^2^Technical Research Centre for Dependency Care and Autonomous Living, Universitat Politecnica de Catalunya, Barcelona, Spain; ^3^Department of Investigation, Consorci Sanitari Alt Penedès - Garraf, Vilanova i la Geltrú, Spain

**Keywords:** wearables, accelerometer, machine learning (ML), Parkinson's disease, medical device

## Abstract

In the past decade, the use of wearable medical devices has been a great breakthrough in clinical practice, trials, and research. In the Parkinson's disease field, clinical evaluation is time limited, and healthcare professionals need to rely on retrospective data collected through patients' self-filled diaries and administered questionnaires. As this often leads to inaccurate evaluations, a more objective system for symptom monitoring in a patient's daily life is claimed. In this regard, the use of wearable medical devices is crucial. This study aims at presenting a review on STAT-ON^TM^, a wearable medical device Class IIa, which provides objective information on the distribution and severity of PD motor symptoms in home environments. The sensor analyzes inertial signals, with a set of validated machine learning algorithms running in real time. The device was developed for 12 years, and this review aims at gathering all the results achieved within this time frame. First, a compendium of the complete journey of STAT-ON^TM^ since 2009 is presented, encompassing different studies and developments in funded European and Spanish national projects. Subsequently, the methodology of database construction and machine learning algorithms design and development is described. Finally, clinical validation and external studies of STAT-ON^TM^ are presented.

## Introduction

Parkinson's disease (PD) is a complex neurodegenerative disorder, presenting a wide range of motor and non-motor symptoms. It is estimated that at least 10 M people have been diagnosed around the world (1), and some studies indicate that this number will keep rising drastically (2). The disease is characterized by different cardinal symptoms (tremor at rest, rigidity, bradykinesia (BKS), and postural instability), as well as non-motor symptoms (3). The detailed and accurate evaluation of the disease is of interest in the management of daily medical practice. Dopamine treatments have been shown to improve motor symptoms and quality of life (4). However, after a certain time undergoing this therapy, some motor complications may appear, such as motor fluctuations (MF), including end-of-dose deterioration (wearing-off) and dyskinesia (DKS) (5), or freezing of gait (FoG). Apart from the motor symptoms, non-motor fluctuations are also present, which make disease management complex (6). It is well-known that MF can appear early in the course of PD. Thus, its early identification is crucial to keeping an optimal quality of life (7, 8). The diagnosis of early fluctuations and dyskinesia is delayed in many cases due to multiple circumstances, such as the short neurology visits and the lack of optimal tools, which can allow for precise symptom identification. This circumstance is also present in advanced stages, where there is also an infradiagnosis of advanced symptoms (9, 10). So far, the identification and quantification of MF can be measured through patient diaries (e.g., Hauser diaries) and/or by a set of validated scales, such as the Unified Parkinson's Disease Rating Scale (UPDRS) (11, 12). Nevertheless, the subjectivity and cognitive state of the patients play an important role in the results. Furthermore, the interrater and intrarrater variability of the UPDRS is significant, leading to confusing evaluation results and highlighting this method's subjectivity (13, 14). On the other hand, Hauser diaries require a great effort and major time consumption from the patients' side. Furthermore, reduced compliance, recall bias, and patient fatigue are also variables to take into account when the Hauser diary is set as a clinical endpoint (15).

Symptoms' evaluation during consultancy results is complicated. The average clinical visits occur around every 6–9 months with about 20 min of time duration (16). Considering this scenario, clinicians are faced with major difficulties in detecting patients who need special care or concrete therapies. In addition to this, medication intake usually happens before going to the doctor's visit, and therefore, real symptoms are not shown in front of clinicians. Thus, the anamnesis performed by the clinician tends to be quite subjective as the symptoms' distribution information is mainly obtained from the patient's point of view. Furthermore, the white coat effect and the Hawthorne effect (behavioral change due to awareness of the patient by being evaluated) affect the symptoms' severity presented at the clinical visit, consequently affecting the whole assessment of healthcare professionals, too (17). Thus, this scenario results in incorrect therapy prescriptions, so decreasing patients' quality of life (QoL). Hence, objective home and daily symptoms monitoring is the key to better understanding the patient's symptoms severity in real life and therefore, prescribing the correct therapy.

Recently, the introduction of targeted tools such as wearable sensors in clinical practice has provided a new approach to collecting motor symptoms in real environments during long-term monitoring in a more precise and objective manner. This new paradigm enables the neurologist to observe clinical symptoms without depending on subjective methods, which come with self-perception bias, or third parties' evaluation, resulting in a non-adequate knowledge or training (18). Furthermore, due to the new social scenario of COVID-19, patients have difficulties and barriers to accessing healthcare facilities and maintaining the usual relationship with their medical service. These technologies allow the patients to overcome these obstacles by being remotely monitored and continuing their relationship with the clinician. Thus, wearable medical devices can become of great support for neurologists to manage movement disorders, especially motor symptoms associated with PD, and consequently improve the QoL and drug treatment of patients (19, 20).

In literature, there have been many approaches to evaluate PD motor symptoms with wearable systems. First, it is important to define the ON and OFF states as the levodopa-related response (5). The ON state is associated with a good levodopa response, while the OFF state is when symptoms re-emerge. One of the most important symptoms that represent an OFF state in PD is bradykinesia. According to Jankovic et al., this is the most characteristic clinical feature of PD (3). Bradykinesia is characterized by slowness in movements, and, in consequence, affects general movement, such as gait. Gait is possibly the best characteristic where a bradykinetic patient can be differentiated from a medicated-ON patient. In a patient affected with bradykinesia, gait is altered by reducing the cadence and the step length, a part of having problems of instability. Bradykinetic gait is affected by levodopa (21, 22), and some studies have focused on bradykinetic gait as a crucial symptom to analyze the behavior of motor fluctuations along the day (23, 24). Due to the motor complications of the disease, the patient takes the medication before the doctor's visit for reaching the healthcare center without mobility problems. However, when the patients take their medication, it hides the main symptoms of bradykinesia, making it difficult to determine the actual condition of the patient.

Another major symptom to be assessed in PD is FoG. This symptom is considered the fifth cardinal symptom of Parkinson's disease (25) and is defined as a “brief, episodic absence or marked reduction of forwarding progression of the feet despite the intention to walk” (26–28). There is an evident correlation between falls and FoG, which leads to a need to accurately treat the symptom (26). FoG is a key symptom for determining a correct therapy prescription, and as some patients do not respond well to levodopa, they need to have a comprehensive evaluation. Given the difficulty to elicit a FoG episode in clinical environments, Nonnekes et al. propose an algorithm for the treatment of FoG and finally, suggest as a solution the use of wearable systems (29).

FoG is very difficult to understand, although several conclusions have been drawn by the scientific community. There are specific conditions where FoG is elicited. This symptom is usually shown in patients with an OFF state, although, in some cases, it can also appear in the ON state (30). The fact that FoG is context-dependent is the reason why it is very difficult to assess it in clinical practice. Thus, in order to measure and quantify this symptom, the freezing of gait questionnaire (FoGQ) (31) and the new freezing of gait questionnaire (NFOG-Q) (32) were designed. Nevertheless, there are some discrepancies in rating FoG with different scales (33), and the NFOG-Q seems to be unsuitable as an outcome in clinical trials according to some experts (34). In this last work, the authors also claim that the use of objective tools such as wearable is essential thanks to their usefulness.

In another study, the issue with FoG assessment in current clinical practice is pointed out. Mancini et al. provide three main arguments in this regard (35). Firstly, FoG disappears while patients walk focusing on goals provided by the clinician. Gait improves when patients consciously focus on walking rather than performing automatic gait. FoG occurs in home environments and real living conditions, not in clinical practice, where the patient is assessed while being observed by a healthcare professional. Secondly, clinical environments are often free of obstacles, not being a space where it is easy to provoke a FoG episode. Thirdly, patients tend to go to the clinical evaluation subsequent to medication intake or in the ON state. The latter affects the physician's evaluation as patients with PD tend to suffer FoG in the OFF state, or, at least, more severe episodes. Mancini et al. claim that wearable systems will be crucial for accurate FoG monitoring.

Regarding PD tremors, it needs to be considered that they differ in types and that not all of them have a dopaminergic response (36). This means that, in many patients, tremor is not correlated with motor fluctuations and can often appear in the ON state. Although there is evidence that some types of tremors are responsive to dopaminergic therapies (37), the same work also remarks that it is not effective for other types of tremors. Thus, tremor monitoring motor state detection (ON or OFF) is often unclear or confusing. Nonetheless, as tremor is manifested in the upper limbs, certainly, wrist-worn devices are a good solution for this symptom evaluation.

On the other hand, levodopa-induced dyskinesias are motor complications caused by the continuous intake of levodopa. These motor complications affect the mobility of the patient, causing involuntary movements in the upper limbs, lower limbs, neck, and trunk. Dyskinesias are related to a decreased QoL (38), as it is a motor complication that should be diminished by adjusting the medication correctly. However, some dyskinesia motor complications are episodic due to the so-called peak-dose dyskinesia, provoked by the L-dopa intake, being difficult to be accurately observed in the doctor's office. In order to evaluate dyskinesia, some questionnaires are administered. However, some articles show that the Unified Dyskinesia Rating Scale is more reliable than other questionnaires (39, 40). Nonetheless, questionnaire administration takes time during consultancy, and, although interrater and intrarrater correlations are moderate according to (40), the questionnaire is administered every so often/occasionally. Among these, the most used questionnaire, the UPDRS, is very dependent on the patient's opinion and does not provide real accurate information about daily symptoms' severity and distribution. This information is key for therapeutic tailoring.

This study aims at presenting a technology solution, which meets the clinical needs of filling the aforementioned lack of objectivity in patients' data in order to quantify the PD motor symptoms during regular ambulatory activity and non-controlled conditions. We present STAT-ON^TM^: a medical device Class IIa based on a single wearable system and able to monitor, measure, hold in internal memory, and finally, generate a report on the temporal evolution of motor symptoms in daily living conditions. First, the state of the art on wearable systems for monitoring PD motor symptoms is presented. Subsequently, related scientific background and assessment of STAT-ON^TM^ in clinical trials, pilots, and algorithm validation processes are explained. Then, the STAT-ON^TM^ system is described, encompassing hardware and software descriptions. Finally, a set of performed clinical validations performed so far is presented.

## State of the Art

There are multiple initiatives and research works on the identification of motor symptoms (13, 14, 41–46), where accelerometers are the most widely used sensors, although gyroscopes (47), skin conductivity systems (48), electromyography (49), pressure insoles (50), and pressure platforms are also used, such as GaitRite (51, 52). Unfortunately, many of these investigations or solutions do not reach the market, mainly due to three barriers: firstly, the poor usability due to a lack of portability of some of these systems, thus making it difficult to incorporate into the daily life of the patient as they are not wearable; secondly, the necessary industrialization process; and, thirdly, the required certification process as a medical device. These last two factors are long, complex, and expensive processes. As mentioned, the most extended solutions are based on inertial systems (47, 53–56), but at present, there is no complete and definitive solution yet. It is necessary to advance in the investigation and development of methods focused on the following points:

- The medical device must provide reliable information from algorithms that have been designed with rigorous methodologies;
- Key and clear information for the healthcare professionals must be presented;
- The design must be focused on the usability of the system in order to maximize the patient's adherence.

The reliability of a monitoring system mainly depends on the following aspects: the number of sensors used, their position on the body, and the robustness in the design of the employed processing algorithm. Brognara et al. (57) state that a trade-off between the number of sensors and the usability of a system should be required. Li et al. (58) also report that a high number of sensors complicate the setting up of a study. Several sensors cause difficulties in synchronizing data, following a timestamp. Furthermore, the number of input features in a machine learning algorithm is increased, consequently increasing the computational burden. However, sensors placed on different parts of the body capture a clearer signal from specific movements and contribute to a better characterization of a symptom (59).

Many of the existing systems for monitoring PD-related motor symptoms rely on a supervised machine learning approach. Algorithms are developed through a learning process based on a specific and representative database. In this way, the dataset employed to build an algorithm is of crucial importance. The dataset must be representative of the problem looking to be solved, and, for this reason, it must be labeled by clinical experts and well synchronized with raw data of the sensors (60). The number and variety of patients with PD that participate in the dataset construction are also the key for the learning of the machine learning method. A good generalization means having a representative dataset, without exceeding the number of patients included, which will introduce noise or repetitive information, but including as many different patients as possible for the generalization of the algorithm against a new input (61).

Apart from increasing the sensitivity or capacity to correctly detect a symptom, having a large heterogeneous database also minimizes the number of false positives and, thus, improves the specificity of a classifier. This is the main reason why a dataset also needs data that do not contain the symptom to be detected. The algorithm will learn what is not the symptom with the aim of maximizing its specificity. Therefore, the experimental protocol must be formed by activities that elicit a symptom so that the raw data (the signal) contain parts with the target symptoms to be analyzed and, also, parts where other activities are included. To do so, the appropriate method is to construct the dataset in home environments, where unforeseen conditions are continuously present (62). Once the dataset is constructed, specific and key features will be extracted and selected from the data and a random part of the data will be used for training the classifier; the other part of the dataset will be used for validating the model. Then, a supervised machine learning technique will be applied (neural networks, support vector machines, etc.), and an automatic classifier model will be obtained that will be cross-validated against the evaluation dataset.

On the other hand, providing key information for the professionals is essential for acquiring an accurate state of motor complications. A medical device should provide information that healthcare professionals do not have in consultancy, that is, the severity and time distribution of motor symptoms in home environments. The report obtained has to be easy and quick to read, embedding self-explanatory graphics. One of the main targets is to increase the usability of the system for healthcare professionals to facilitate a dynamic patient's visit with quality information on key symptoms. In (46), some graphical examples of different commercialized devices are shown. On the other hand, usability for patients is also essential. Usability will define the patient's adherence to the use of the technology. Wrist-worn devices have been shown to be devices prone to very high usability (63). They are comfortable, and the patient does not feel stigmatized. However, in order to analyze bradykinetic gait, freezing of gait, or dyskinesia (which is manifested in upper and lower limbs, trunk, or neck), an inertial wrist-worn device is not able to capture accurately these symptoms, and other devices would be better, such as the waist or chest-worn devices from where body movements are better characterized due to being close to the center of mass of the human body. The main issue with wrist-worn devices is the high degrees of freedom of movements made by the arm, in addition to their high randomness of execution, provoking an elevated rate of false positives, causing a decrease in the specificity (64). Several studies point to serious errors in this type of system for the characterization of steps or momentum (65, 66).

Taking a look into the global market, there are, at least, four commercialized tools with a Medical Certification (CE Certificate with the Directive 93/42/EEC or with Regulation 2017/745, FDA, or other regional certificates, such as CFDA, TFA, etc.) able to monitor Parkinson's disease symptoms: Personalized Kinetigraph™ (67), Kinesia 360™ (68), PD Monitor™ (69), and STAT-ON™ (70). There are other solutions, such as MM4PD (55) or NEPTUNE (71); both are wrist-worn devices in different stages of technology readiness but, still, without medical certification. Furthermore, so far, no clear evidence or article has been published on the algorithm methods used in these last two devices. In the review performed in (72), different algorithms are also proposed as techniques to be embedded in hardware solutions in order to detect motor fluctuations. Other solutions focus more on gait, which can be also interesting (73–78), but they do not provide continuous monitoring at home with a complete mapping of the different symptoms of PD.

Table 1 presents a comparison between the aforementioned identified solutions, including the list of the different symptoms monitored by each solution.

**Table 1:** Parkinson's disease continuous monitoring systems.

**Manufacturer**		**Global Kinetics Corporation**	**Great Lakes Neurotech**.	**Pdneuro-Technology**	**Apple**	**Orbit Health**	**Sense4Care**
**Device name**		**PKG**^®^ **or Kinetigraph**^®^	**KINESIA360**™	**PDMONITOR** ^®^	**MM4PD**	**NEPTUNE**	**STAT-ON**™
**Device Location**		**1 wrist sensor**	**2 sensors (wrist/ankle)**	**5 body-worn sensors**	**1 wrist sensor**	**1 wrist sensor**	**1 waist sensor**
Detected symptoms	ON/OFF	Yes	Yes	Yes	No	No	Yes
	Bradykinesia	Yes	Yes	Yes	No	Yes	Yes
	Dyskinesia	Yes	Yes	Yes	Yes	Yes	Yes
	Tremor	Yes	Yes	Yes	Yes	No	No
	Freezing of Gait	No	No	Yes	No	No	Yes
	Gait Parameters	No	Yes	Yes	No	No	Yes
	Inactivity/Rest	Yes	Yes	Yes	No	No	Yes
	Falls	No	No	No	No	No	Yes
Medical device certification		Yes	Yes	Yes	No	No	Yes

An important point is the analysis of the algorithms developed by each manufacturer and the validation performed. While PD MONITOR™ and STAT-ON™ are based on advanced machine learning techniques, Kinesia360™ bases its algorithms on multiple regression methods and PKG™ on a statistical analysis of two variables.

PKG™'s algorithm was published in 2012 by Griffiths et al. (79). The authors presented a method based on the analysis of the accelerometer signals obtained from the wrist during 2-min windows. From this window, they analyzed frequency features between 0.2 Hz and 4 Hz, the maximum acceleration achieved, and the time without movement, from which the two indexes are generated. One is associated with bradykinesia (BKS), and the other with dyskinesia (DKS), which are then represented in a chart with interquartile ranges to then determine the severity of a symptom or the other. There is no evidence of a training-evaluation data method, thus not being considered a machine learning algorithm. The validation was performed through the median of all the BKS samples in 9 h along 10 days and was correlated with the UPDRS, obtaining a significant *r* = 0.64, *p* < 0.0005. A third score called FDS was designed to measure motor fluctuations (80). This score, which is expressed as an algebraic combination of BKS and DKS, determines whether a patient is fluctuating. However, the method cannot determine the “ON” state without dyskinesia (81). Although there are no data that confirm the performance of an algorithm with blind data, the device has been widely tested under clinical conditions and compared to UPDRS (82) or diaries (80, 81). For instance, the work from Santiago et al. (83), determines that PKG™ provides more information to classical routine visits after analyzing 3 user cases. In a work performed by Nahab et al., the authors also denote utility in clinical practice (84). Finally, the system has shown good results in usability (85). According to Monje et al. (13), the PKG™ has been extensively validated but needs more independent validation.

Kinesia 360™ is another device to monitor Parkinson's disease. The algorithm is more complex than the PKG™ one and uses a gyroscope to add information value. The system is composed of two sensors, a wrist-worn device, and an ankle-worn system, which, on the one hand, obtains information about gait. The latter is crucial to understanding the state of a patient with PD; nonetheless, the dual system reduces usability for the patient.

Kinesia 360™ offers outcomes from tremor, dyskinesia, slowness, mobility, posture, and steps (68). The quantification of bradykinesia, which was performed in an ankle-mounted device (86, 87), relies on the analysis of some specific features coming both from the accelerometer and gyroscope and is computed through linear regression models, which are correlated with the UPDRS scores. The dyskinesia algorithm is also based on a linear regression model, and the sensors are worn on the most affected side of the body. The correlation obtained is significant (*R* = 0.77) and is performed with the modified Abnormal Involuntary Movement Scale (mAIMS). The models are evaluated through a Leave-One-Subject-Out method. The system has been widely evaluated with different therapies, such as levodopa (53), rotigotine patch (88), deep brain stimulation (89), or subthalamic stimulation (90).

Finally, PDMonitor™ is a five-device system in which the main aim is to characterize all the motor symptoms of a patient with PD coming from any part of the body. In this way, it is not necessary to select the most affected side of the body, and it is possible to get movements from the upper limbs, lower limbs, and trunk. The system was designed in the PERFORM project (46), and its algorithms are based on the training of an expert database and using advanced machine learning algorithms. The complete system is presented in (47), and the algorithms are briefly described, such as tremor (91), dyskinesia (92), bradykinesia (93), and FoG (94). All the algorithms employ different classification methods. For instance, tremor is based on hidden Markov models, obtaining an 87% of accuracy; dyskinesia algorithm is based on a decision tree, reaching 85.4% on classification accuracy. The bradykinesia algorithm uses support vector machines, with a 74.5% on classification accuracy, and the FoG algorithm relies on a random forest classifier, getting a significant accuracy of 79%. Although the PERFORM project is well documented, and the algorithms are transparent, as far as the authors know, there is no evidence that the system has been validated in clinical practice.

In summary, it can be understood that it is not possible to directly compare the four devices, given the different locations in the body, the number of sensors, or the algorithmic used methodology (learning-based or statistical-based). The only work found so far with a direct comparison between devices is a work from Grahn, which compares the agreement between PKG™ and STAT-ON™ with 2 physicians (95). The agreement between the clinical opinion and STAT-ON™ was found to be significantly higher than PKG's™; on the other hand, both devices show to be usable by patients. Although STAT-ON™ shows superiority in this work, further studies are needed with more consistent data.

The following section presents a compilation of the methodology used in the case of the STAT-ON™ solution.

## Background on Stat-ON^TM^-Embedded Algorithms

The STAT-ON^TM^ device is the result of a long research process and development based on different and complementary achievements gathered in several research projects managed and participated by the authors. The base of the algorithms to detect and monitor the relevant PD motor fluctuations relies on gait parameters analysis, complemented with another set of specific algorithms dedicated to the identification of concrete symptoms and characteristics: bradykinesia, dyskinesia, FoG, detection of falls, or the signal magnitude area (SMA), for the assessment of the quantity of movement.

A starting point for this activity was the publication in 2009 of the hypothesis about the possibility of adjusting the necessary dose of apomorphine pumps by detecting motor fluctuations with wearable sensors (96). Continuing with the study of motor symptoms in PD, new lines of research, focused on ambulatory monitoring of specific motor symptoms, were mainly performed in the projects *Monitoring the Mobility of Parkinson's Patients for Therapeutic Purposes* (MoMoPa Project) (97), *Home-Based Empowered Living for Parkinson*'*s Disease* (HELP project) (98, 99), *Personal Health Device for the Remote and Autonomous Management of Parkinson*'*s Disease* (REMPARK project) (24, 100, 101), the MoMoPa-2 project (102), and the MASPARK project (103). Within these projects, the resultant algorithmic set was validated by introducing new patients. Finally, once a consistent clinical validation was achieved, *Unobtrusive, Continuous, and Quantitative Assessment of Parkinson*'*s disease: Hard Evidence for Optimal Disease Management with Information Technologies* (PARK-IT2) project (104) was performed in order to redesign the existing prototype, embed the developed algorithms, industrialize and certify it as Medical Device Class IIa. The final device was considered clinically usable by a group of neurologists (54), and it is being validated in different pilots and clinical trials (105, 106).

At the starting point of the described research, a preliminary decision was considered on the number of sensors to be used and their location in the body for optimal detection of the PD motor symptoms, along with an optimal usability characteristic. After analyzing different parts of the body, the waist was selected, given that it is very close to the mass center of the body and many movements are reflected there in some way. This situation provided very clear inertial signals from the gait, upper and lower limbs movements, and trunk or neck dyskinesia. Concerning the number of used sensors, the decision was very clear, and the objective was to use a unique sensor located in the waist, as has been mentioned.

Following this decision, a coherent and strict methodology was developed, including a very complete analysis of the gait. Initial gait parameter algorithms were achieved by selecting specific features using accelerometer signals from the waist by combining them with different kernel methods (107). This algorithm was improved in posterior research projects, such as MoMoPa-2 (102), or MASPARK (103) by improving the methodology for gait characterization, focusing on the bradykinetic gait (108, 109). The estimation of bradykinesia severity relies on a specific methodology mainly based on the detection and characterization of gait. Several filters were implemented, and the first one is formed by a Support Vector Machine classifier (SVM), which detects if the patient is walking or not by analyzing specific features, which have been selected by means of the Relief algorithm. The detection of walking is followed by the detection of strides in terms of walking bouts. This stride is then characterized with different features in order to linearly separate “*bradykinesia*” from “*no bradykinesia*” through a threshold β, whose value is set by means of an ϵ-Support Vector Regression (ϵ- SVR) model with RBF kernel (110, 111). The ϵ- SVR model depends on a set of parameters extracted from stride fluidity (*m*): the mean, standard deviation, minimum, maximum, and median. Other inputs of the ϵ- SVR model are the Hoehn and Yahr stage and the age of the patient, which are factors that show the advance of the disease and limit the movement fluidity of the patient. All these seven variables will be inputs of the ϵ- SVR model whose output is the threshold β. The ϵ- SVR model is then trained and evaluated following a Leave-One-Subject-Out methodology. Results obtained show an average sensitivity of 0.925 and 0.891 of specificity, with an accuracy of 0.918 on bradykinetic gait detection (109).

As it is described in (112), a self-adapting bradykinesia detection algorithm is incorporated. The threshold β enables the recognition of bradykinesia in terms of ON and OFF. For instance, a young patient without motor complications provides a high β threshold; however, a patient with advanced-staged PD with motor complications would provide low β values. Therefore, ON and OFF states are patient-dependent, and first, the algorithm needs to understand and learn the stage of the patient. To do so, and considering the input variables for the ϵ- SVR algorithm, a concrete patient self-adapted algorithmic part was developed, requiring minimum information from the person's movement in order to establish the correct parameters. It has been stated that 3 days of monitoring are enough to get enough stride fluidity values and to learn how the patient walks, fluctuates, and behaves within his or her motor fluctuations. From the 3rd day on, the healthcare professional could obtain the data and see the ON and OFF state in the downloaded data. If the data are downloaded on the 5th day, it means that the β value has been computed with these 5-day data. The main obtained advantage of the inclusion of this part is the minimization of the external parameters to be manually introduced and a new evaluation of the necessary threshold β for every new use of the device. This allows the reuse of the sensor among different patients and allows easier disease evolution monitoring.

The main goal of the algorithmic set, as established in the REMPARK project, is the identification and registration of the ON and OFF states of affected people. The final decision to determine an ON or OFF state is conditioned to the sustainability of this β threshold along time. Another crucial factor for the decision of the ON and OFF algorithm is the detection of levodopa-induced dyskinesia symptoms, which considerably increases the probability of establishing an ON state.

Dyskinesia algorithm was first designed in (113), where frequency power was extracted in the considered frequency band of dyskinesia (1–4 Hz), defined by Manson et al. (114), and, also, analyzing the frequency band up to 20 Hz in order to remove false positives if the patient was walking. The algorithm was simple, but thresholds were optimized by maximizing positive predictive value and negative predictive value. The algorithm was improved significantly by using machine learning in the optimization of thresholds and other features, such as the inclusion of the postural transitions' frequency band (115). The final model was not patient dependent, being general and valid for any patient with PD. The database used to train and validate the algorithms was composed of 102 patients. The presented algorithm showed a performance of 0.39 on sensitivity and 0.95 on specificity on mild dyskinesia, but a 0.93 on sensitivity in any strong dyskinesia and trunk mild dyskinesia, keeping the 0.95 on specificity. This specific work was performed in the frame of the REMPARK project (24). In the same framework, the algorithm was validated clinically against the Unified Dyskinesia Rating Scale (UDysRS) (40), considering the severity of the dyskinesia. The algorithm correlated 0.7 with all the UDysRS questionnaires, but the correlation increased up to 0.91 when only sub-items from the UDysRS were considered for dyskinesia in the trunk and legs (116).

The developed ON/OFF algorithm is a hierarchical structure of classifiers that get together the outcomes of specific algorithms, such as the bradykinesia and dyskinesia, and observe the behavior at regular slots of time (1, 10, and 30 min). The output data rate of the ON/OFF algorithm provided by STAT-ON^TM^ is precisely 30 min. The complete explanation of this algorithm is given in (112). However, a third state was introduced and was called “*Intermediate*.” This state stands for that motor state where the patient is not walking in his or her better condition, but the stride fluidity is better than his or her OFF state.

In REMPARK's project, this proposed ON/OFF algorithm achieved a 0.92 both on specificity and sensitivity (112). This study contains the presentation of the methodology to detect the motor fluctuations, and the results were compared to a specific Hauser diary. The patient had to fill in the Hauser diary, but a researcher performed a supervision call to the patient every 2 h to confirm the motor state in order to maximize the confidence of a correct diary annotation. This algorithm was then validated against the opinion of direct observers, UPDRS (117), and Hauser diaries (118). In a work by Rodriguez-Molinero et al. (119), 20 patients participated in a database from which the algorithm model was trained, following the methodology explained in the aforementioned work by Pérez-López et al. (112). The algorithm model was then validated by employing 15 new patients, and the results of the algorithm were compared against the opinion of trained observers who stayed with the patients the whole time during the validation test. The results obtained were 0.96 on sensitivity and 0.94 on specificity, showing significant robustness.

In another published work from Rodriguez-Molinero et al. (20), the ON/OFF algorithm was validated against UPDRS subscales (UPDRS-III), with the participation of new 75 patients with PD. The correlation with all UPDRS-III was moderate, achieving a rho = −0.56 (*p* < 0.0001); however, the correlation with the gait item increased to 0.73 *p* < 0.001, and a correlation with Factor I item on UPDRS (axial function, balance, and gait) was −0.67 (*p* < 0.01), considered as a significant correlation. The algorithm was also validated against Hauser diaries to compare the method with other gold standards.

In a work performed by Bayes et al. (19), a total of 41 patients with PD participated in a 3-day test, where the patients were asked to fill in the Hauser diary. In this experiment, and with the aim of having rigorous control, the researchers called the patients in order to verify their motor state. Only when the result of the diary and the call were equal, then it was considered “comparable” to the sensor. This condition elevated the rigorousness of the Hauser diary, given the reduced compliance and recall bias that this method uses to present (15). A total of 0.97 on sensitivity and .88 on specificity were achieved following this method.

Finally, the ON/OFF algorithm was also validated against the Hauser diary in (120), where a total of 23 patients participated. One of the most important conclusions was to realize that a total of 37% records more were achieved by the sensor in the pilot, showing the reduced compliance obtained with the diaries. Also, it must be noted that, in these experiments, clinicians tried to minimize the rejection rate by filling the diaries by administering MoCA or MMSE questionnaires. This fact is the key because the patient does not need any interaction with the sensor. In this study, the accuracy (0.92) was provided along with positive (0.92) and negative (0.94) predictive values.

**Figure 1:**
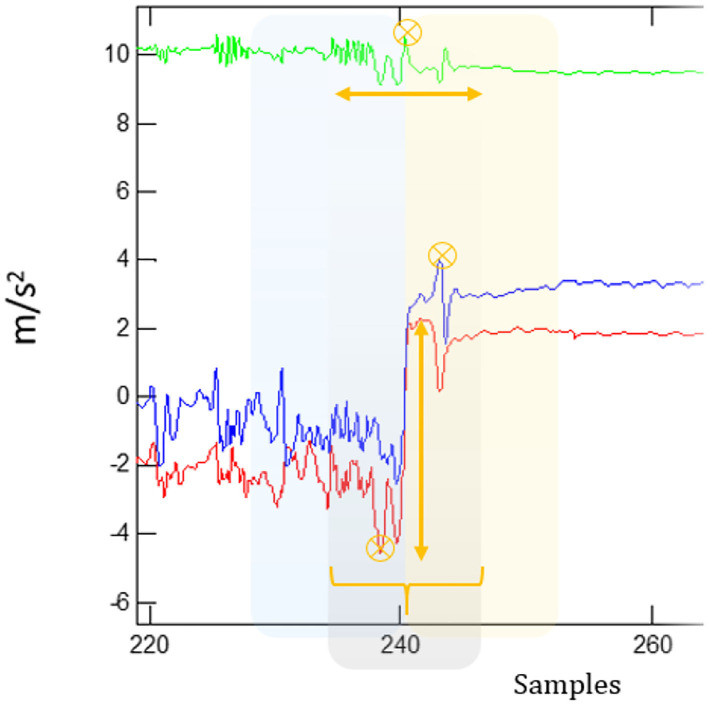
Windowing of a signal at 50% overlapping and feature extraction.

Complementing the ON/OFF, bradykinesia, and dyskinesia algorithms, the FoG algorithm was also embedded within STAT-ON^TM^. The algorithm is based on a machine learning approach based on SVM (62). The database was performed in home environments with 21 patients performing semi-guided activities in ON and OFF states. The fact that data were collected at each patient's home provoked different situations and FoG episodes in their real daily living activities, not in clinical settings. All the FoG episodes (except the akinetic ones) were video-recorded and labeled by experts. The inertial signal associated with this label and the generated database was used for training the algorithm with supervised machine learning methods, including SVM. The generated algorithm was evaluated through a strict method, which balanced the true negative episodes, which could be given in long-term activities where FoG episodes were not possible to appear, such as sleeping, sitting, or standing still. This detail is the key and showed a more reliable specificity compared to other evaluation methods. Specificity depends on true negatives and false positives. If a true negative was considered as the evaluation of the algorithm every second, then in 30 min, we would have 1,800 true negatives, falsely increasing the specificity of the algorithm, although there were 10 false positives in those 30 min. Thus, we only counted a single true negative episode every 30 s, giving a more realistic specificity in this concrete time frame. The classifier designed analyses, filters, and processes 3.2 s-windowed signals overlapped at 50% with the aim of not losing information that occurred between windows (Figure 1). Then, specific features are extracted from each window, and a set of characteristics is organized by assigning a label *y*_*w*_ for each *w* window.

The *y*_*w*_label was set to “*1*” if that window contained a FoG episode regardless of its length. For instance, if the FoG episode was 1-s long, then that window was considered to have a FoG episode and was labeled as “*1*.” If the window did not contain any FoG episode, then it was labeled with a “*-1*” value (62).

Each window contains a total of 55 features, which were employed as an input of an SVM classifier in which the used kernel was a Gaussian radial basis function (RBF) due to its good performance and generalization capacity. Following this method, it was achieved a general classifier model with 0.75 on sensitivity and 0.79 on specificity for the detection of FoG episodes. However, the method was then improved by applying a feature selection and deleting noise (121). In this work, several classifiers, such as logistic regression, neural networks, or SVM with different hyperparameters, were tested, and it was shown that SVM with RBF kernel worked better with optimal resources. Finally, the new and optimized method achieved a 0.92 on sensitivity and 0.87 on specificity and was compared in the same conditions with other published methods, showing a significant improvement. The embedded algorithm was evaluated by clinicians with 12 patients in (122), where a 0.82 on sensitivity and a 0.97 on specificity were achieved. The model was not self-adaptive, being general for all the patients.

Additional gait parameters, such as stride fluidity, step length, cadence, and stride speed, are obtained based on the algorithm presented by Sayeed et al. (108), where 28 patients with PD participated, and an accuracy of 0.96 was obtained in the detection of gait. One of the most important patient characteristics is energy expenditure or the quantity of movement. The STAT-ON^TM^ provides the quantity of movement through the Signal Magnitude Area (SMA parameter), which was first tested in the sensor in (123), employing the accelerometer signals in the 3 axes to analyze the variability of the signal in a concrete period.

On the other hand, the STAT-ON^TM^ also incorporates an algorithm to detect falls; the algorithm, which was tested in the FATE project for a whole year with 200 patients, was embedded within the device (124, 125). The fall algorithm achieved 0.95 on sensitivity and 0.99 on specificity. Finally, a postural classifier and posture transition algorithm is incorporated in order to achieve specific information about the patient's activity (123, 126).

The conditions and results obtained in each algorithm embedded within STAT-ON^TM^ are presented below in Table 2. In Year/Project column, the project is presented from which the data were trained and validated. *M1* stands for MoMoPa-1 (97), *M2* stands for MoMoPa-2(102), *M3* stands for MoMoPa-3 (127), *RE* stands for the REMPARK project (101), *MA* stands for MASPARK (103), and *SP* stands for specific expert databases.

**Table 2:** A summary of the main results obtained in the included algorithms.

	**References**	**Year/project**	**Evaluation reference**	**Number of patients**	**Evaluation result**	**Result**
ON/OFF algorithm	Pérez-López et al. (112)	2016/M2	Hauser diaries with patient calls every 2 h	15	Sensitivity/specificity	0.92/0.92
	Rodriguez-Molinero et al. (119)	2015/M1	Hauser diaries with patient calls every 2 h	35	Sensitivity/specificity	0.94/0.96
	Bayes et al. (19)	2018/RE	Hauser diaries with patient calls every 2 h	41	Sensitivity/specificity	0.97/0.88
Bradykinesia estimation	Samà et al. (109)	2017/M1,M2	Video recording	12	Sensitivity/specificity	0.925/0.891
			UPDRS subscales		Pearson correlation	UPDRS (item 22) : −0.912; *p <* 0.001
					Pearson correlation	UPDRS (item 24): −0.808 *p* < 0.001
					Pearson correlation	UPDRS (Factor I): −0.834; *p* < 0.001
	Rodriguez-Molinero et al. (20)	2017/RE,M2	UPDRS subscales	75	Spearman correlation	UPDRS (part III): −0.56; *p* < 0.001
						UPDRS (Item 22): −0.73; *p* < 0.001
						UPDRS (Factor I): −0.67; *p* < 0.01
Levodopa induced dyskinesia	Pérez-López et al. (115)	2016/RE	Video recordings	102	Sensitivity/specificity	No-trunk, mild dyskinesia: 0.39 / 0.95
						Trunk, mild dyskinesia: 0.78 / 0.95
						No-trunk, strong dyskinesia: 0.90/0.95
						Trunk, strong dyskinesia: 1 / 0.95
	Rodriguez-Molinero et al. (116)	2019/RE,M3	UDysRS	13	Spearman correlation	UDysRS score: 0.70; *p* = 0.01
						UDysRS sub-item (trunk and leg): 0.91; *p ≤* 0.001
Freezing of Gait	Rodríguez-Martin et al. (62)	2017/RE,MA	Video recordings	21	Sensitivity/specificity	0.75/0.79
	Samà et al. (121)	2017/MA	Video recordings	15	Sensitivity/specificity	0.92/0.87
	Rodríguez-Martin et al. (122)	2017/MA	Video recordings	12	Sensitivity/specificity	0.82/0.97
Gait	Sayeed et al. (108)	2015/RE	Video recordings	28	Accuracy	0.96
Falls	Cabestany et al. (124, 125)	2013/SP	Patients' case report forms	205	Sensitivity/specificity	0.95/0.99
Postural transitions	Rodríguez-Martin et al. (123)	2013/M1,SP	Video recordings	39	Sensitivity/specificity	0.86/0.98
	Rodríguez-Martin et al. (126)	2015/RE,SP	Video recordings	87	Sensitivity/specificity	0.90/0.91

## Stat-ON^TM^, The Holter for Parkinson's Disease Motor Symptoms

### The STAT-ON^TM^ Hardware

**Figure 2:**
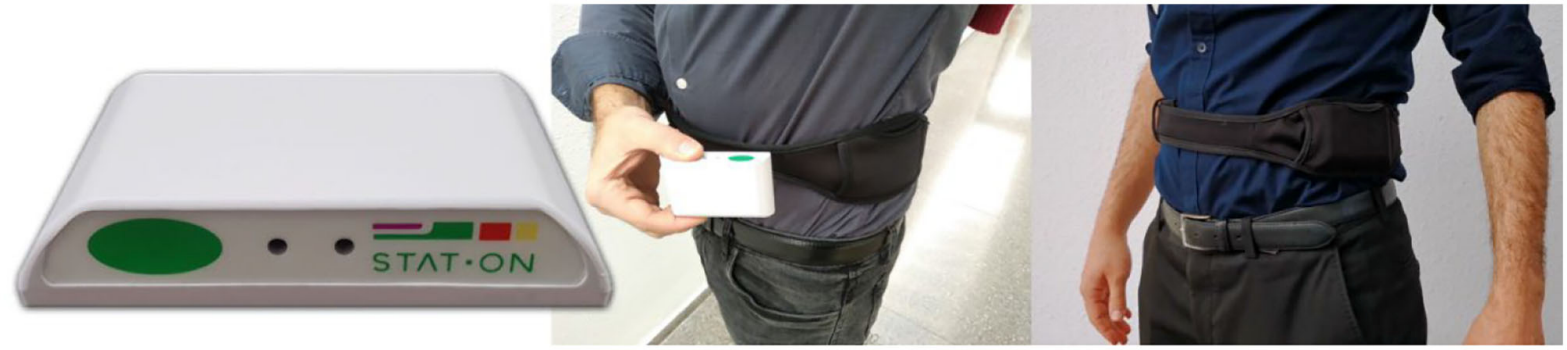
STAT-ON^TM^ and its location and orientation.

STAT-ON^TM^ is an inertial wearable medical device Class IIa. Concretely, the STAT-ON^TM^ system consists of a monitoring device as shown in Figure 2, a base charger, a belt, and a mobile application. The system provides numerical and graphical information of the motor symptoms' presence and distribution associated with Parkinson's disease based on a real-time processing embedded version of the algorithms referred to in Section Background on STAT-ON^TM^-Embedded Algorithms. Furthermore, data related to the general motor activity of the patient are also computed according to the concepts introduced in the precedent Section Background on STAT-ON^TM^-Embedded Algorithms.

The sensor measures 90 mm^3^ x 62.5 mm^3^ x 21.2 mm^3^ and weighs 86 grams. Internally, the system is composed of two ultra-low triaxial nano-accelerometers, two microcontrollers, and a Bluetooth Low Energy system, among other parts. The sensor has a battery life of 7 days for a continuous operation in normal conditions (8 h per day). The system is waterproof with IP65 protection. The enclosure is formed by two pieces that fit each other by a specifically designed sealing strip, which is included for waterproofing purposes. The material selected for the enclosure is POLYLAC ® FR-ABS, an acrylonitrile butadiene styrene (ABS) material. Some of the main features are flame rated, RoHS compliant, and heat and weather resistant.

**Figure 3:**
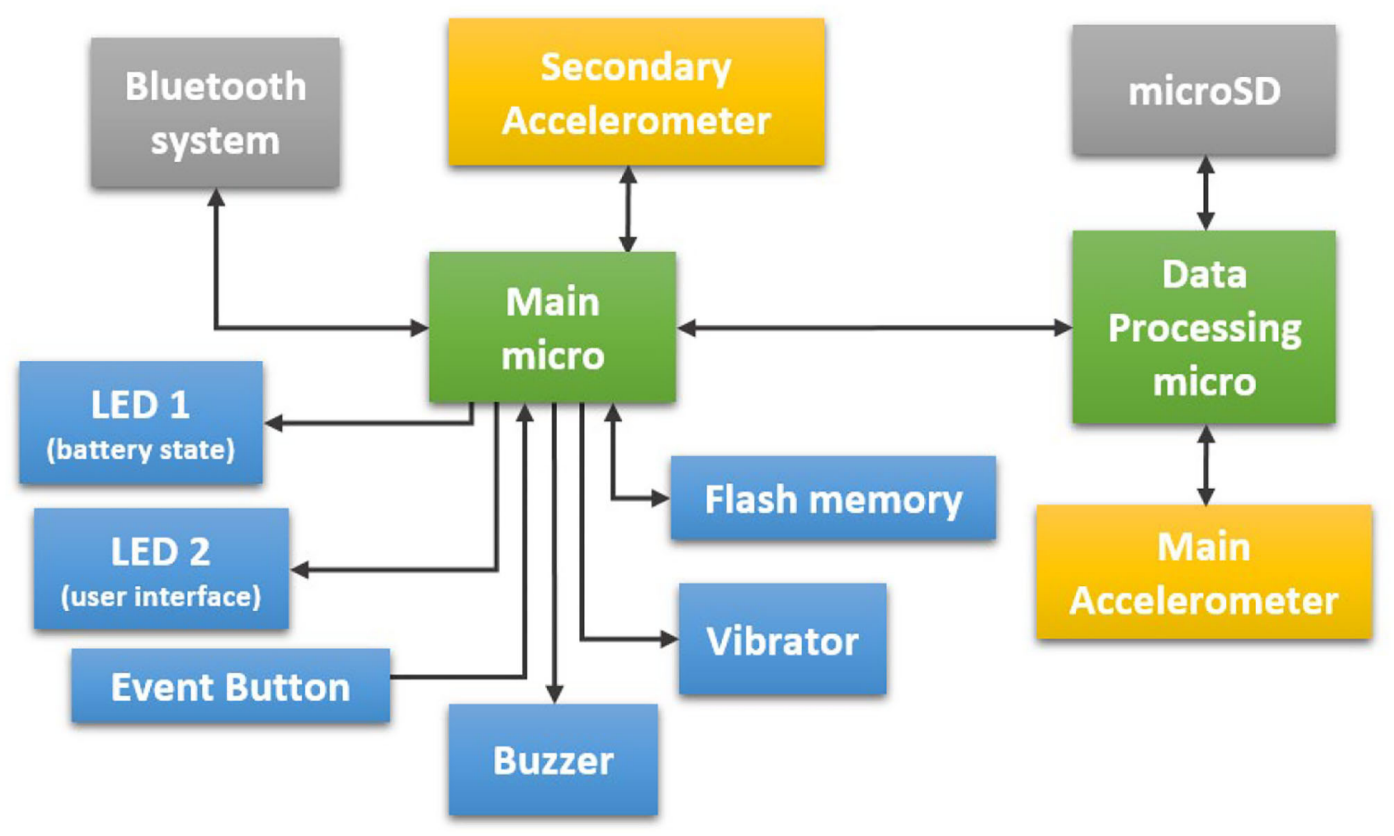
STAT-ON^TM^′s internal structure.

As shown in Figure 3, the sensor is formed of two microcontrollers: the main one is an nRF51822 that manages all the internal processes of the system and that has incorporated internally a Bluetooth (BLE) system (128). The second microcontroller is the STM32F415 microcontroller, which has a Cortex™ M4 core (with a floating-point unit) running at 168 MHz for operating complex mathematical models, such as the SVM classifiers, or the signal filtering and featuring required by the described algorithms (129).

The main microcontroller manages the user interface (LEDs, event button, buzzer, and vibrator), and stores the outcomes of the algorithms in the internal flash memory. This microcontroller also manages the states of the medical device, such as the sleeping state in case of a lack of movement, or active state in case the patient is performing some movement. The necessary flags for the definition of these conditions are provided by the secondary accelerometer, which detects the absence of movement or wakes up the system in case of movement detection (128).

The communication part of the system is based on Bluetooth Low Energy (BLE) and is used only when the clinicians configure the system or when the healthcare professional requires the downloading of the data monitored and internally stored after the processing phase. The microcontroller STM32F415 is responsible for computing all the inertial signals provided by the main accelerometer, an LIS3DH that provides a 50-Hz signal to the microcontroller. In parallel, the system provides the possibility to store the raw complete data from the accelerometer inside a microSD card.

The system includes a vibrator and a buzzer to send alarms to the patient or caregiver, such as medication reminders, which can be configured with the STAT-ON^TM^ app. The user can also find the event button, whose target is to indicate a concrete event. This event will be registered internally and will be shown in the graphs generated by the STAT-ON^TM^ app. There are two LEDs: the first one indicates the state of the battery (charging or not), and the second provides different color codes to inform the user of the state of the system, such as “connected,” “capturing data,” “low battery alarm,” “error alarm,” “synchronizing,” and “configuring.”

**Figure 4:**
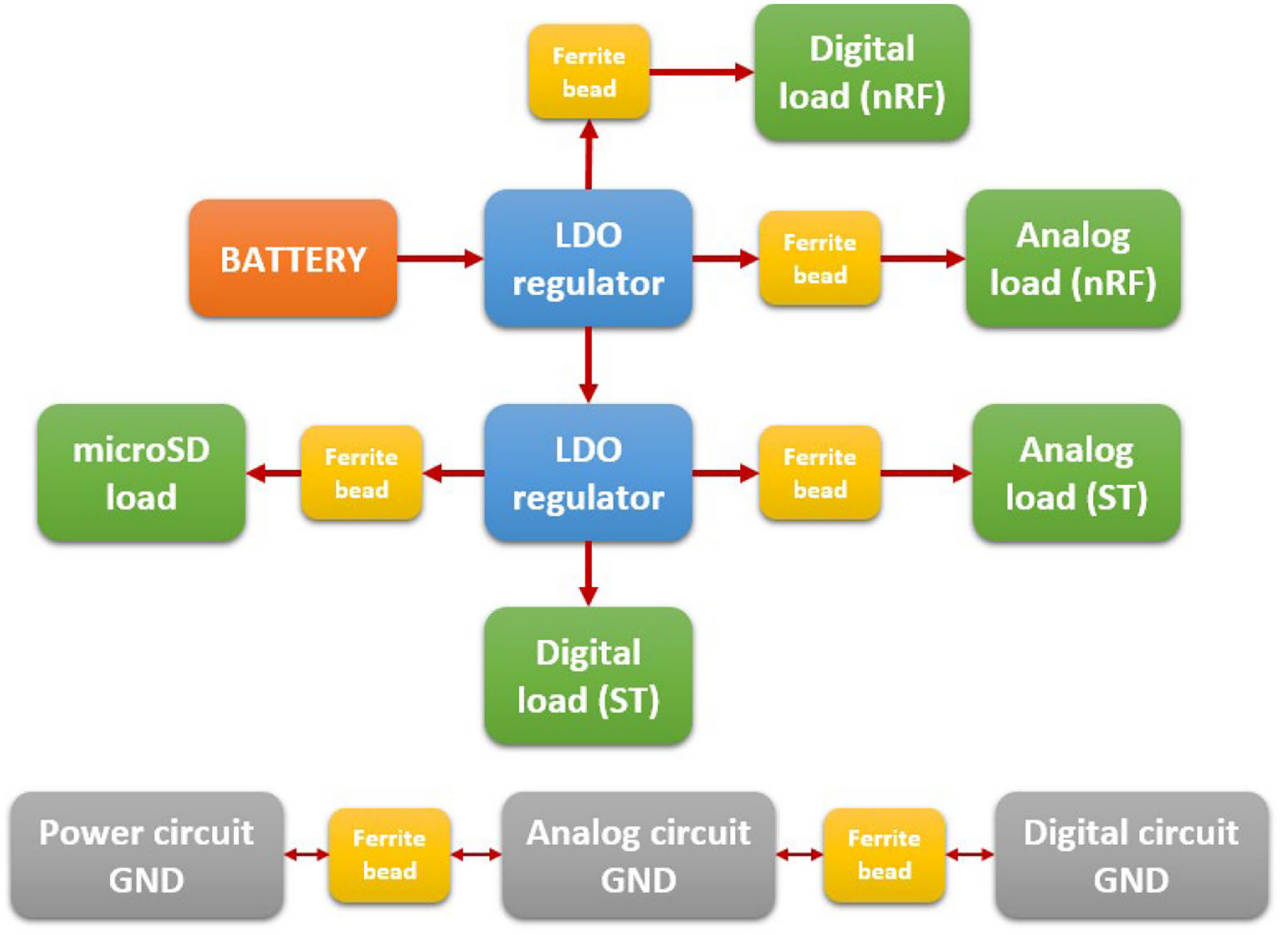
Power system and regulator managing.

The management of power consumption is very important. For this reason, the power system is divided into three separated electrical zones: analogic, digital, and power system zones. Different regulators manage each zone, being isolated by specifically designed grounds and ferrite beads, as shown in Figure 4.

The power system includes a fuel gauge (BQ27441) for controlling the voltage level and managing the battery. Also, it includes the BQ51050B, a Qi-compliant wireless power receiver with an integrated Li-Ion/Li-Po battery charge controller. The power system is connected to a specific coil that sets the communication with any commercial wireless Qi-compliant chargers in order to charge the device wirelessly.

The system has been certified as Medical Device Class IIa and has successfully passed the electromedical equipment tests under IEC60601-1, including the EN ISO 60601-1-11 for home environments use. The device is manufactured under ISO 13485 for medical devices. The software explained in the next section has been certified under EN 62304 for medical software.

### STAT-ON^TM^ Software

In the frame of the project PARK-IT2 (104), where the redesign and industrialization phases of STAT-ON^TM^ were done, the algorithmic set described in Section Background on STAT-ON^TM^-Embedded Algorithms was completely embedded in the aforementioned hardware, and a new software layer for the management, interfacing, and correct usability of the sensor was created. A specific app is required to be installed on a smartphone/tablet, which is the current operative interface with the user (a healthcare professional).

The regular use of the STAT-ON^TM^ consists of wearing the device in home environments with the aim of capturing activities of daily living of the patient and the fluctuations of the disease, as well as the severity and frequency of PD's motor symptoms. Firstly, the healthcare professional, with a specific smartphone app, will configure the device with just three parameters crucial for the algorithms of walking and bradykinesia estimation: age, H&Y stage, and leg length. Then, the device is provided for the patient, who should wear it during wakening hours and in normal conditions (the sensor must not be used while taking a shower, traveling, or doing sports except hiking) for 5–7 days approximately. After the monitoring period, the healthcare professional will download, using the same smartphone/tablet app, all the outcomes computed and stored by the sensor. These outcomes are organized as a complete report of the activity and the symptoms' presence and their evolution. The healthcare professional can also decide to download a more complete report generated by the device, where details are more explicit, together with comprehensive gait information during the monitored period.

**Figure 5:**
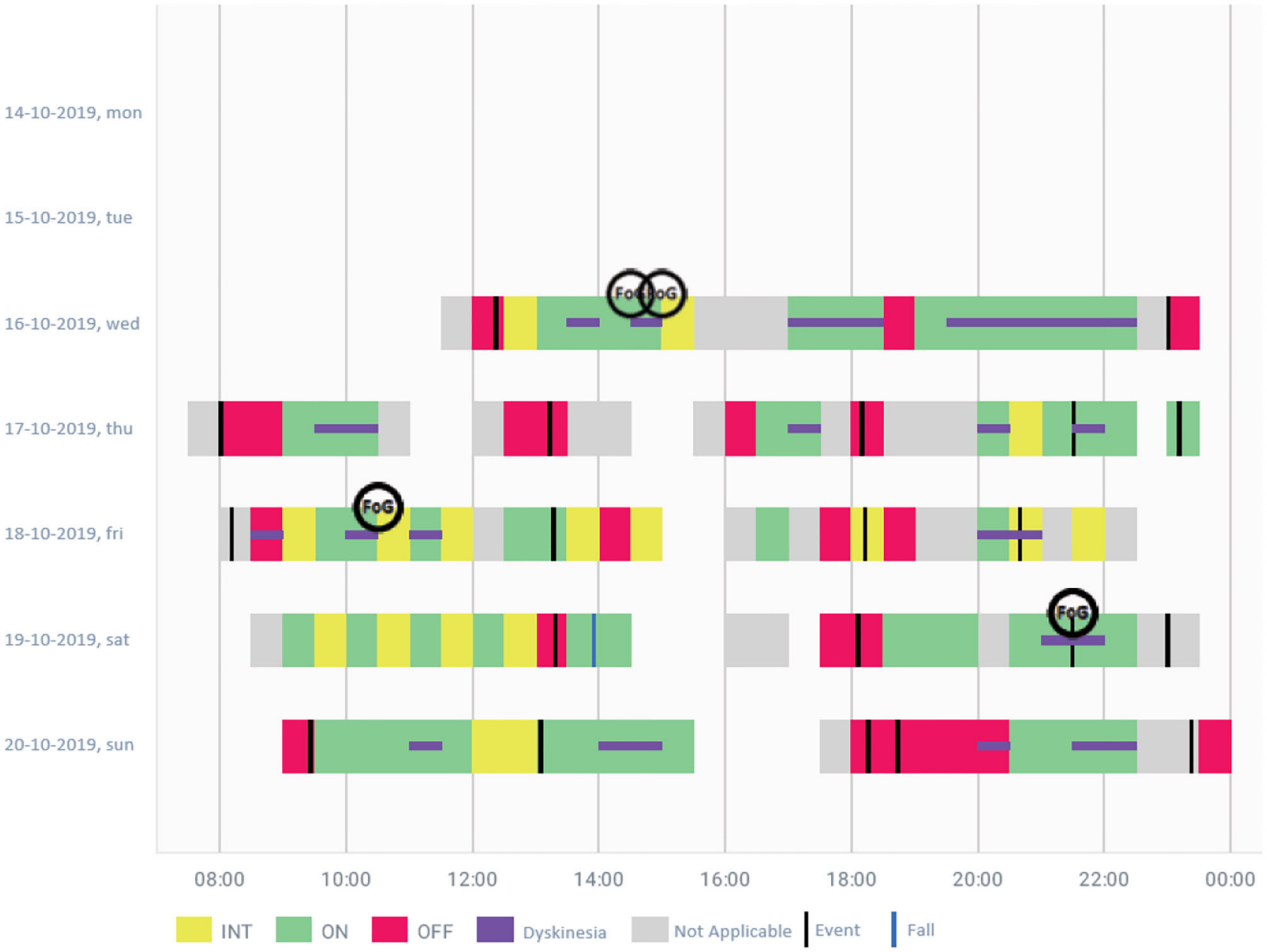
An example of the distribution of symptoms (hourly distribution of symptoms along different days following the established color code), one of the graphics generated by STAT-ON^TM^. This patient has a concurrent OFF zone every day around 18:00. It is clear when the patient rests at 15:00 or 16:00 every day. The OFF and dyskinesia are significant, but the FoG only appears 4 times, being practically insignificant and should be contrasted with the patient. The black line is when the button was pushed, in this case when the patient took the medication.

Data are presented with different graphs with a fixed temporal resolution of 30 min and 24 h for weekly data graphs. The structure of the report consists of the first page, reporting the summary of the monitoring period, and then the distribution graph is presented (Figure 5).

**Figure 6:**
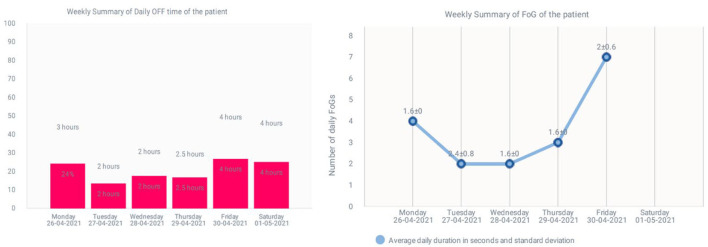
An example of the percentage of OFF hours and total OFF hours and the number of FoG episodes per day.

The software offers the possibility to download a basic report with the previous graphs and also with quick information about the percentage of OFF hours per day and the total amount of OFF hours per day. Finally, a graph reporting the number of FoG episodes per day is presented. An example of both graphs is shown in Figure 6.

For an extended analysis, there is the possibility to obtain an extended report with the rest of the information. Then, weekly graphs are shown followed by detailed daily graphs of each variable. The values presented in the temporary format of 24 h are: cadence, number of steps, step length, SMA (quantity of movement), stride fluidity, dyskinesia, ON state, OFF state, INT state, number of FoG episodes, duration of FoG episodes, falls, and events generated through the sensor button.

**Figure 7:**
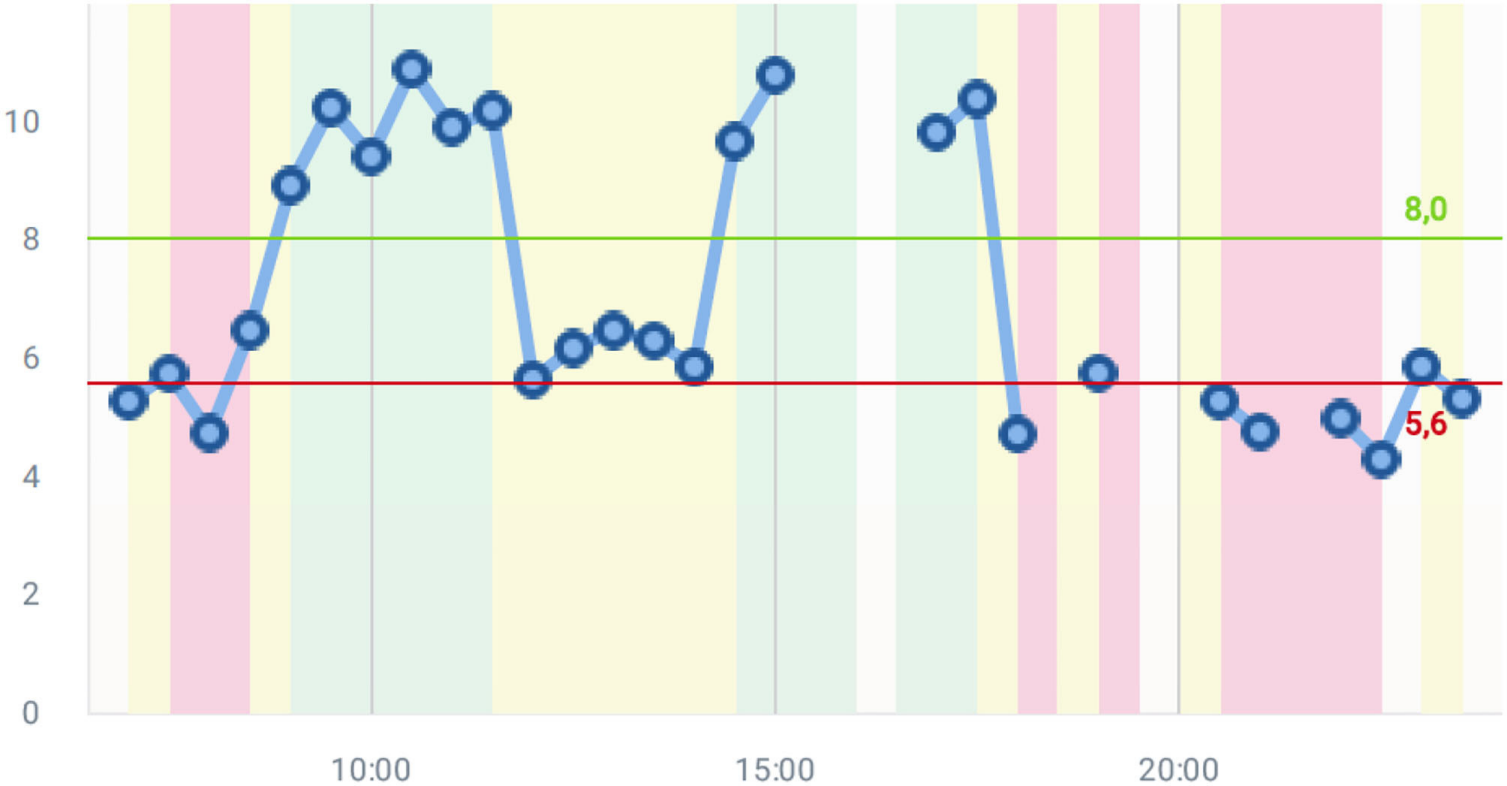
A stride fluidity example.

Figure 7 shows the stride fluidity graph, which is one of the most important graphs and offers the severity of the bradykinetic gait. Scores obtained in this graph are based on the algorithm of bradykinesia estimation (109). In this case, it is shown a patient with fluctuations passing from ON to OFF and inversely. Two clear OFF zones are detected in the morning and the evening. At midday, the patient has low scores, but the OFF seems not to be very significant. The objective information creates a quick picture of the state of the patient. In this Figure, the two thresholds are determined after having learned how the patient walks for 3 days based on the self-adaptive algorithm described in Section Background on STAT-ON^TM^-Embedded Algorithms.

**Figure 8:**
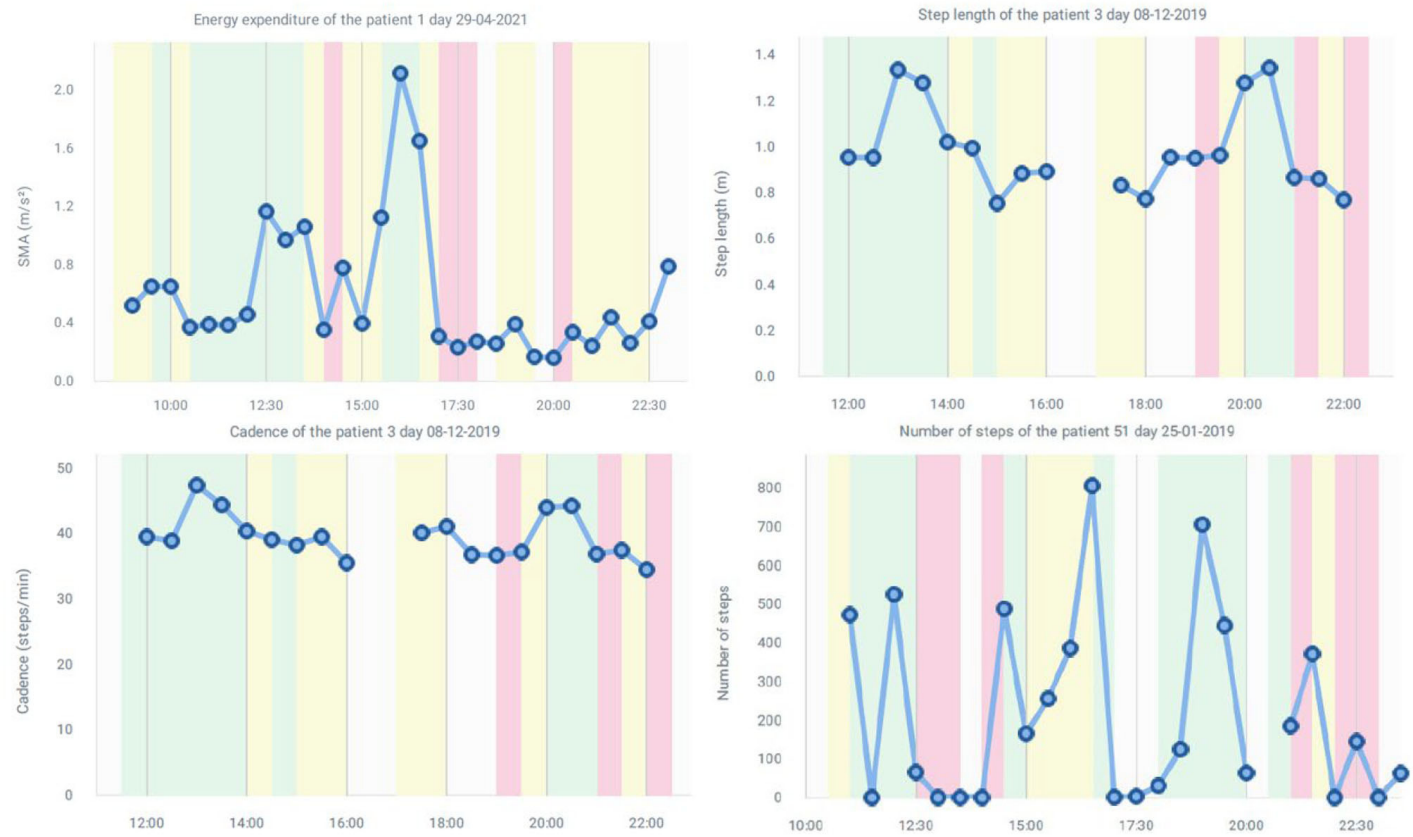
Some graph examples of the extended report.

Figure 8 shows possible graphs provided by STAT-ON^TM^, such as energy expenditure, step length, cadence, or the number of steps. It must be noted that more information is provided, such as stride speed, weekly summaries, and detailed daily motor states (70). Given that these graphs are filtered every 30 min, for more detailed information and for research purposes, it is better to use the CSV file, with the detailed information per minute. The graphs are mainly used in clinical practice.

## Clinical Validation of Stat-ON^TM^

STAT-ON^TM^ started its commercialization in June 2019, when the CE mark was obtained. It was then that the validation (from the regular clinical praxis point of view) of the commercialized device started. So far, the device has been validated in several studies.

A series of questionnaires were performed within the PARK-IT2 project in order to understand the acceptability of the device (130). A total of 107 questionnaires were performed, involving 17 neurologists, 19 health professionals, 30 caregivers, and 41 patients. A significant 88% of neurologists thought that STAT-ON^TM^ was able to detect advanced PD symptoms, and the average score of the sensor was 7.9/10. Healthcare professionals gave a score of 8.6/10 to the sensor. On the other hand, 80% of caregivers found STAT-ON^TM^ a good or very good solution and no one disliked the sensor. Moreover, 76% thought that it was easy to use, and no caregiver reported the belt was difficult to wear and adjust. The patients also rated the sensor an 8.5/10, and 77.5% thought that it was very easy to use. The belt was rated 8.1/10.

In 2020, Santos et al. published the opinion of 27 clinical experts in movement disorders about STAT-ON^TM^ after having tested the device in clinical practice (54). The general opinion of the neurologists was promising and with some important conclusions. A total of 119 evaluations were performed with different patients with PD using a STAT-ON^TM^ sensor. In conclusion, STAT-ON^TM^ was considered better than diaries by 70.3% of neurologists, and it was also considered a useful tool to detect advanced Parkinson's disease by 81.5% of the involved neurologists. The device was considered “quite” to “very useful” by 74% of the participants, and a moderate correlation between the use of the sensor and the opinion of the physician was obtained (*r* = 0.403; *p* = 0.046). A total of 89% of neurologists would use STAT-ON^TM^ in their clinical practice.

A clinical trial has been proposed to test the device against other considered gold standards, such as the Hauser diary and the UPDRS (106). This clinical trial is a single-blinded randomized controlled trial. The neurologists who participated in this study were randomly assigned to one of the three branches of the study in which a therapeutic adjustment would be performed based on different sources of information: the STAT-ON^TM^ reports, the patient diary of motor fluctuations, or the clinical information collected at the consultancy.

A total of 162 patients were participating in this study for 6 months, and the main outcome is to compare the efficiency of STAT-ON^TM^ against classical clinical practice methods in terms of OFF-time reduction. Other symptoms will be also evaluated, such as dyskinesia and FoG, and the non-inferiority of the sensor against the diary of motor fluctuations will be also evaluated (https://clinicaltrials.gov/ct2/show/NCT04176302) (106).

On the other hand, a pilot is being led by the Movement Disorders Unit, “UParkinson” from Centro Médico Teknon, Grupo Hospitalario Quirón in Barcelona, Spain (105). The pilot consists of analyzing the agreement in detecting motor fluctuations, dyskinesia, and FoG using the STAT-ON^TM^, based on a patient's opinion, and a neurologist's opinion in the home environment. The first preliminary results showed that the sensor can increase the awareness of motor fluctuations in patients with PD and help healthcare professionals detect them earlier. The level of satisfaction (QUEST questionnaire) achieved significant results (all items over 4 out of 5). The System Usability Scale (SUS) questionnaire results were considered high.

In another study, which was presented at the Annual Meeting of the Spanish Neurology Society, Caballol Pons et al. (131) discussed a multi-centric work, considering a high number of STAT-ON^TM^ reports (in concrete, 237) in different use cases. The most frequent reason given by the neurologists for using STAT-ON^TM^ was the ON/OFF time quantification, followed by the detection of FoG/falls and dyskinesias. The device is being used in patients with both initial and advanced PD for the diagnosis of motor complications and/or treatment optimization.

Due to the COVID-19 pandemic, telehealth systems are also important tools to be considered, and STAT-ON^TM^ meets the requirements to be classified as a helpful system for the remote monitoring of patients with PD. Currently, the device is used in a clinical trial where patients with PD are being monitored remotely with video calls and STAT-ON^TM^ (https://clinicaltrials.gov/ct2/show/NCT04694443).

In an Argentinian study, a team of neurologists tested the device with 11 patients and reached some interesting conclusions. The comparison against diaries showed that the Holter registers were bigger than diary registers, showing one of the main issues of the diary: low patient compliance. Also, the study highlights the enhanced patient's awareness of FoG episodes, as the sensor detected them while the patient did not report them. The sensor information was useful as neurologists could see objectively the real behavior of FoG episodes. It is also important to note that the authors emphasize the importance of the sensor in guiding therapeutic decisions in clinical practice. This was reported in patients who need second-line therapies, and the decision is based on questionnaires and the doctor's office evaluation. Finally, the authors conclude that these tools were useful to obtain an objective measure of the patients' motor state who were in advanced stages of the disease, with difficulty controlling motor symptoms, inconsistencies in their daily reports, and suspicion of inappropriate medication intake (due to lack or excess medication) (132).

The device has been also validated with advanced-stage PD patients with levodopa-carbidopa intestinal gel. Bougea et al. demonstrated the better detection of ON/OFF motor fluctuations, dyskinesia, and falls against patients' diaries with 51 patients with PD. All the sensitivities and specificities were higher with the sensor rather than with the diary, concluding that STAT-ON^TM^ can be a promising tool for monitoring patients with advanced disease (133).

STAT-ON^TM^ was also used in patients that were administered PERCEPT™, a deep brain stimulator that also registers the signal perceived from the subthalamic nucleus field, remarkably aligning their signals in the appearance of OFF states, ON states, dyskinesia, and FoG episodes. This case study suggests that STAT-ON^TM^ can be a useful tool for the optimization of this kind of therapy (134).

Finally, in a Swedish study, STAT-ON^TM^ was tested and compared against PKG™ through resident physician criteria. A significant agreement was obtained between STAT-ON^TM^ and the physician (kappa = 0.783, *p* = 0.014), and none was found between PKG and the physician (95).

## Conclusions

Currently, technology offers multiple possibilities for interaction and monitoring of patients with chronic diseases. In the field of PD, the main drawbacks are the lack of objective information obtained by the physician and the fact that the consultancy or hospital is not the most convenient environment for a correct patient evaluation. They should be evaluated, when possible, in normal living conditions in their home environments.

There are wearable tools that provide objective information about the severity and distribution of PD motor symptoms that could improve the evaluation of clinical experts. However, all devices in the market have their pros and cons. The strongest point of STAT-ON is, undoubtedly, the accuracy of the algorithms, which have been designed with precise data obtained in home environments and with a sensor located in a very specific part of the body, very close to the center of the human body. The waist has been shown to be akin to human movement, and also many movements can be characterized from there. However, this strong point could be also a weak point, given that the usability that a wrist-worn device will always be higher due to the lower invasiveness of the device. Nevertheless, the devices that are worn on the wrist are conditioned to the random movements in the arm that should be considered for maximizing specificity. This point is of key importance to get high accuracies, and this problem has not been already solved. On the contrary, these devices are socially accepted and might be very useful for obtaining approximate measurements of basic movements.

Concerning other sensors, there has to be a trade-off between the number of sensors, usability of the wearable system, and the accuracy of a system. In this paper, we presented a complete review of STAT-ON^TM^, a wearable medical device that provides objective information on motor symptoms, such as bradykinesia, dyskinesia, ON-OFF fluctuations, FoG, and gait parameters, falls, the quantity of movement, and postural activity. The purpose of use of this device is focused on home environments in order to get the missing data, which a healthcare professional cannot obtain in his or her consultancy. A complete review of the algorithms is performed, opening up the possibility to improve the outcomes by combining different machine learning approaches, enlarging the database or using deep learning or other more advanced methods.

The clinical evaluation in real clinical practice with STAT-ON^TM^ has already started, although the first results have been achieved by having a great acceptance rate by different stakeholders: patients with PD, caregivers, neurologists, and healthcare professionals. The utility and acceptability in clinical practice are promising (54), and, although further research and validation should be carried out, results show the potential of an easy-to-use tool. The STAT-ON^TM^ has achieved great results in user satisfaction and usability (105) and has been used in many cases (131), such as detection of motor fluctuations, dyskinesia, freezing of gait, therapy optimization, or second-line treatments' patients' selection. Nonetheless, further studies are needed for early symptoms detection and to demonstrate the effectiveness of the device with different therapies. However, it seems that there is a consensus on using the device for the detection of candidates for second-line therapies (54, 132). The Antonini et al. consensus for the selection of patients for advanced therapies seems to align with the outcomes of STAT-ON^TM^, but additional findings are required to confirm this (135). The fact that the main database in REMPARK was composed of patients with fluctuations and Hoehn & Yahr >2 in ON state (24), the algorithms have been focused on mid and advanced stages of PD. This is particularly beneficial as a tool for an appropriate selection of the patient for second-line therapies and for adjusting these therapies (continuous subcutaneous apomorphine infusion, levodopa-carbidopa intestinal gel, or deep brain stimulation). However, a challenge is to see if STAT-ON^TM^ works fine for earlier stages. In the work performed by Caballol et al. (105), they detected morning fluctuations, which are the first OFF episodes in early fluctuating patients, but more studies are needed. One of the limitations of STAT-ON^TM^ is that the ON-OFF algorithm does not work in patients who are unable to walk, and 3 days of data are necessary for learning the way the patient walks. The accuracy of the ON-OFF algorithm has been shown but takes too much time to achieve results. Also, according to the user manual, it is recommended to use the device between 5 and 7 days (70). Although the patient can wear the device for more time, it is enough between 5 and 7 days to see patterns, severity of symptoms, and their distribution.

Another point and limitation is the understanding of the FoG algorithm. In (54), the FoG algorithm was considered one of the weaker algorithms. However, it has to be taken into account that this algorithm output is given every 1.6 s, while the ON-OFF is given every 30 min and the possibility to provide a false positive increases. The specificity presented in (121) is 0.87, which is considered optimal. However, some false positives could appear in festinating gaits, by tripping, traveling by car or public transport, and doing sports. Nevertheless, the device could identify properly the FoG in ON and OFF states, and help the healthcare professionals to understand this symptom in patients with PD as was shown by Perrote et al. (132).

The utility of wearable devices is increasing widely in the field of PD. In a Spanish study discussing the future of Parkinson's evaluation, 94% of 75 experts in movement disorders think that the use of wearables will increase (136). The conjunction of complement devices is a topic of discussion for achieving the best evaluation of patients with PD. For example, non-motor symptoms detection, such as depression, anxiety, fatigue, orthostatic hypotension, and sleep disturbance, have not been investigated deeply (137, 138). Nevertheless, there are some approaches for sleep disorders, such as electroencephalograms and eye tremor analysis (139). Also, heartbeat and blood pressure (140), or even skin conductance (141), have been used for non-motor symptoms. All these systems need further evaluation and more studies. In the same line, telemedicine is also a future challenge, and pilots and further tests are needed to validate the system for this purpose, which is crucial in post-pandemic times.

Another point is the use of the achieved data to continue improving the algorithms: several machine learning techniques will be published in the future, and even additional data could be obtained through STAT-ON^TM^ in future projects for refining the algorithms based on the opinion of physicians.

According to the first hypothesis performed by some of the authors in 2009 (96), STAT-ON^TM^ and other monitoring devices could be used in a closed-loop system to automatically adjust the therapy; this idea is closer to the appearance of new medical devices but needs accurate devices, with well-validated algorithms both in computer science and medical journals and in controlled clinical trials.

STAT-ON^TM^, which is a marketed medical device, is the result of 12 years of research, including algorithmic development based on machine learning and offering a complete solution in clinical practice, trials, and research. The device can be used for adjusting and personalizing therapies, selecting patients for specific therapies, following up on specific symptoms, and seeing objectively the severity and distribution of PD motor symptoms. Although more validation is needed in the future, the system has been shown to be useful for healthcare professionals and suggests a new paradigm in the clinical evaluation of patients with PD.

## Author Contributions

DR-M and JCab: conceptualization, methodology, and project administration. DR-M, CP-L, and MP: hardware and firmware. MP, AS, and CP-L: software. CP-L and MP: validation. DR-M, CC, and JCab: formal analysis. DR-M, CP-L, and AS: investigation. DR-M, CC, AR-M, AC, and CP-L: resources. DR-M and CP-L: data curation. DR-M: writing the original draft preparation. CC, JCab, CP-L, MP, JCal, AC, and AS: writing, reviewing, and editing. CC, DR-M, JCab, CP-L, and MP: visualization. JCab, CC, AC, and AR-M: supervision. JCab, DR-M, and JCal: funding acquisition. All the authors have read and agreed to the published version of the manuscript.

## Funding

This study has been funded by the European Commission, Project No. 756861, PARK-IT2.0. H2020-SMEINST-2-2016-2017 and REMPARK FP7-ICT-2011-7-287677. The MoMoPa series of projects have been funded by the Spanish Instituto de Salud Carlos III. The Maspark project was funded by LaMarató de TV3.

## Conflict of Interest

DR-M, JCab, CP-L, AC, AS, JCal, MP, and AR-M are shareholders of Sense4Care, the company that markets STAT-ON. DR-M, JCal, MP, and CC are employed by Sense4Care.

## Publisher's Note

All claims expressed in this article are solely those of the authors and do not necessarily represent those of their affiliated organizations, or those of the publisher, the editors and the reviewers. Any product that may be evaluated in this article, or claim that may be made by its manufacturer, is not guaranteed or endorsed by the publisher.
